# Are social determinants associated with depression among married women of reproductive age? A mixed methods study from urban slums of Islamabad, Pakistan

**DOI:** 10.1371/journal.pgph.0003463

**Published:** 2024-07-23

**Authors:** Muhammad Ahmed Abdullah, Babar Tasneem Shaikh, Nargis Yousuf Sattar, Balaj Sarwar, Ameer Sikander Ahmed, Syeda Sara Fatima

**Affiliations:** 1 Health Services Academy, Islamabad, Pakistan; 2 Professor of Public Health, Health Services Academy, Islamabad, Pakistan; 3 Central Institute of Family Medicine, Islamabad, Pakistan; 4 Dr. Akbar Niazi Teaching Hospital, Islamabad, Pakistan; 5 Shifa College of Medicine, Islamabad, Pakistan; NYU Grossman School of Medicine: New York University School of Medicine, UNITED STATES OF AMERICA

## Abstract

Depression among married women of reproductive age is on the rise in Pakistan, owing to post-COVID-19 phase, super-inflation, increasing poverty, deteriorating law and order situation and perpetuating the uncertain political situation in the country. This study aimed to investigate the factors associated with depression among married women of reproductive age in Pakistan, using a mixed methods approach. The quantitative phase utilized the Urdu version of the Patient Health Questionnaire-9 (PHQ-9) to assess depression among 340 married women. Twelve women with higher scores on the PHQ-9 were selected for in-depth interviews in the qualitative phase. The quantitative analysis revealed a higher prevalence of depression among women with poor socioeconomic status, lower educational levels, larger family sizes, and recent deaths in the family. In the qualitative phase, the main themes identified were the impact of social conditions, such as societal expectations and gender roles, the influence of medical conditions on mental health, financial difficulties, the stress associated with caring for a larger number of children, and the emotional burden of recent deaths in the family. This study highlights the importance of addressing depression among married women of reproductive age in Pakistan. It is crucial to focus on early diagnosis and prompt treatment to mitigate the adverse effects of depression on the affected individuals and their families. Targeted interventions should consider the social determinants of depression, including improving socioeconomic conditions through safety nets, providing mental health support at the primary health care level, and addressing the specific health issues and needs of women in the reproductive age group. A multi-pronged approach and health system’s thinking can reduce the burden of depression among women, ultimately enhancing their overall well-being, productivity and quality of life.

## Introduction

According to the World Health Organization (WHO), depression represents a greater burden of disease as compared to several other physical illnesses, where one in four people suffer from depression during their lives [1, 2]. Research has shown that depression is linked to increased morbidity and mortality [3]. Depression influences thoughts, feelings, emotions, behaviors, relationships and also affects social role, education, and productivity [4]. Depression is a major determinant behind overall burden of disease, disability and death globally [5, 6]. Depression can significantly impair an individual’s daily functioning, relationships, and education, leading to reduced productivity and quality of life [7]. Despite the availability of various treatment options, depression remains a complex and challenging condition to diagnose and manage [8]. Depression stands as one of the most commonly overlooked and inadequately addressed mental health conditions on a global scale. Therefore, it is one of the mounting public health issues especially among women of reproductive age as they are disproportionately affected by depression, owing to the typical social, cultural and gender dynamics of the developing countries [9, 10].

The occurrence of depression is twice as prevalent in women compared to men. In Pakistan, the prevalence of depression among women ranges from 29% to 66%, while for men, it varies from 10% to 33%. [11]. According to the existing published work, determinants of women health in Pakistan include marital status, social support, inter-spousal communication, educational levels and family type [12]. The reluctance of women to express their emotional, social, and physical needs is also correlated with the presence of depression in women [13]. Past studies have indicated a robust connection between gender discrimination and psychological morbidity among women in Pakistan [14]. Depression was found to be associated with relationship and adjustment difficulties with husbands and in-laws, the number of children, and financial challenges [15]. Furthermore, the death of a loved one and the advancing age of married women are also correlated with depression. On the other hand, social and family support are viewed as buffering agents or protective factors against depression [16]. Depression among married women is on the rise in Pakistan, owing to post-COVID-19 phase, super-inflation and increasing poverty, deteriorating law and order situation and perpetuating uncertain political situation in the country [17]. The main objective of this study was to understand the factors associated with depression among married women of reproductive age. Hence, the present study purports to look afresh into the issue of depression, its triggers, prevalence and its effects on physical and social environments of women from slum/rural areas of Islamabad, the capital city and a rapidly growing metropolitan of Pakistan.

Identifying all potential determinants is crucial for developing preventive strategies to address the prevalence of depression among women. Mental health is mostly neglected component in the health system and hence, the toll of depression is increasing day by day.

## Methods

The study design utilized a mixed methods approach, incorporating both qualitative and quantitative research components. The study was conducted in four Family Medicine clinics over a duration of 8 weeks (March to April, 2022) during which the study participants were recruited. The clinics in Islamabad territory were chosen which are managed by the physicians having post-graduation in family medicine and with interest in mental health of women. These clinics are part of a consortium formed under the Central Institute of Family Medicine, Islamabad. The study participants were recruited following consecutive non-random sampling technique and enrolling those who fulfill the basic eligibility criteria i.e., married women of reproductive age belonging to lower middle and middle income groups. Data was collected by a team of young researchers who were given prior training and an opportunity to pilot the study tools before going into the field.

### Ethics statement

Study was granted the ethical approval from the Ethics Committee of Islamabad Medical & Dental College. Key considerations for protecting human subjects during the research included: i) ensuring privacy during the data collection, ii) confidentiality and anonymity of data, iii) treating study participants with respect and dignity, and iv) taking voluntary written informed consent from all the study participants before the interviews.

### Quantitative component

Following a cross-sectional study design, the quantitative data collection was done utilizing a validated Urdu version of the Patient Health Questionnaire-9 (PHQ-9) [18]. The PHQ-9 is a widely used tool for assessing depression and consists of nine self-administered questions. The scoring system of the PHQ-9 was applied to evaluate the severity of depression symptoms among the participants. The sampling technique employed was purposive sampling, including all consenting married women of reproductive age group (18–49 years). A sample size of 340 participants was estimated through WHO sample size calculator and the formula for specified absolute precision, was utilized. The anticipated prevalence for this calculation was kept at 33%, based on a previous study [11]. The data was analyzed using descriptive statistics and correlation analysis, to examine the prevalence and severity of depression symptoms among the participants.

### Qualitative component

From the participants enrolled in the quantitative component, those who scored significantly higher on the PHQ-9, a subset of 12 women was selected for in-depth interviews. The qualitative interviews were conducted on the principle of data saturation i.e., where data collection did not generate new themes or no new insights emerged from the conversations. In-depth interviews of 40 minutes on average were conducted to explore the experiences, perceptions, and contextual factors related to depression among the selected participants (for questions, see S1 Text). After some rapport building and explaining the goal and objectives of the research in layman language, the interviews were conducted in a private and comfortable setting, allowing for open and candid discussions. The interviews were audio-recorded with the participants’ written consent and later transcribed verbatim for analysis. Qualitative data analysis involved the systematic examination and interpretation of non-numerical data to uncover patterns, themes, and meanings. Using thematic analysis, the transcripts were carefully reviewed, and themes were identified based on recurring patterns, concepts and experiences expressed by the participants. Group coding was done for building broader categories and for developing themes that capture key aspects of the data. The themes related to social conditions, medical conditions, financial situation, number of children, family type, and deaths in the family were identified and analyzed. Researchers ensured to maintain reflexivity in order to avoid own biases and assumptions. For details, please refer to the COREQ checklist (S1 Checklist)

## Results

During the course of this research, we explored depression among 340 women and out of those who scored more than 15 on the PHQ-9 scoring, twelve women were interviewed in-depth. The basic demographics of the study participants are given in Table 1.

**Table 1:** Basic demographics of participants.

Variables	Frequency Distribution	Proportion
**Age**	18–23	65 (19.1%)
	24–29	78 (22.9%)
	30–35	56 (16.5%)
	36–41	48 (14.1%)
	42–49	93 (27.4%)
**Educational Status**	Uneducated	45 (13.2%)
	Can read and write	62 (18.2%)
	Primary school	85 (25.0%)
	Matric	72 (21.2%)
	Intermediate	52 (15.3%)
	Bachelors and above	24 (7.1%)
**Number of Children**	None	25 (7.4%)
	One	68 (20.0%)
	2–4	123 (36.2%)
	5–7	97 (28.5%)
	More than 7	27 (7.9%)
**Area of Residence**	Rural	112 (32.9%)
	Urban Slum	78 (22.9%)
	Urban	150 (44.1%)
**Average Monthly income**	50–100	87 (25.6%)
	100–200	102 (30.0%)
	200–500	98 (28.8%)
**USD**	Above 500	53 (15.6%)

Women’s depression scores were analyzed along with several demographic variables as shown in Table 2. The results revealed that 75 (22.1%) of the respondents had no depression, while 94 (27.6%) suffered from mild depression. Additionally, 82 (24.1%) women were found to have moderate depression, and 63 (18.5%) participants experienced moderately severe depression. Lastly, 26 (7.6%) women were identified as having severe depression.

**Table 2:** Percentage of responses for PHQ 9.

Problem	Not at all	Several days	More than half the days	Nearly every day
1. Loss of interest	250 (73.5%)	70 (20.6%)	14 (4.1%)	6 (1.8%)
2.Feeling down	265 (77.9%)	50 (14.7%)	20 (5.9%)	5 (1.5%)
3.Altered sleep patterns	280 (82.4%)	45 (13.2%)	10 (2.9%)	5 (1.5%)
4.Tiredness and lethargy	230 (67.6%)	70 (20.6%)	30 (8.8%)	10 (2.9%)
5. Altered appetite	260 (76.5%)	60 (17.6%)	15 (4.4%)	5 (1.5%)
6. Self-Worth Impact	210 (61.8%)	70 (20.6%)	45 (13.2%)	15 (4.4%)
7.Reduced concentration	220 (64.7%)	60 (17.6%)	40 (11.8%)	20 (5.9%)
8.Altered responses	275 (80.9%)	40 (11.8%)	20 (5.9%)	5 (1.5%)
9.Suicide/death ideation	305 (89.7%)	25 (7.4%)	10 (2.9%)	0 (0%)

When examining the relationship between depression scores and age, it was observed that among women aged 18–23, 65 (19.1%) had no depression, 78 (22.9%) experienced mild depression, and 56 (16.5%) had moderate depression. In the age group of 24–29, 48 (14.1%) women had moderately severe depression. Among women aged 42–49, 59 (17.4%) had no depression, and 34 (10%) were classified as having severe depression.

Regarding educational status, it was found that among uneducated women, 45 (13.2%) had no depression, while 62 (18.2%) women who could read and write suffered from mild depression. Among those with a primary school education, 85 (25.0%) had moderate depression. Furthermore, among women with a matriculation level of education, 72 (21.2%) experienced moderately severe depression, while 24 (7.1%) women with a bachelor’s degree or above had severe depression.

In terms of the number of children, it was observed that among women with no children, 25 (7.4%) had no depression, while 68 (20.0%) experienced mild depression. Among those with 2–4 children, 123 (36.2%) had moderate depression. Moreover, among women with 5–7 children, 97 (28.5%) experienced moderately severe depression, and 27 (7.9%) women with more than 7 children had severe depression.

When considering the area of residence, it was found that among women living in rural areas, 112 (32.9%) had no depression. In urban slums, 78 (22.9%) women suffered from mild depression, while in urban areas, 150 (44.1%) experienced moderate depression. Women who expressed suicide ideation and exhibited more severe symptoms were referred to the nearest public psychiatric care facility.

Lastly, in terms of average monthly income, among women with an income range of 50–100 USD, 87 (25.6%) had no depression. Among those with an income range of 100–200 USD, 102 (30.0%) experienced mild depression. For women with an income range of 200–500 USD, 98 (28.8%) had moderate depression. Lastly, among women with an income above 500 USD, 53 (15.6%) experienced moderately severe depression.

The following themes emerged during the qualitative data analysis, of the 12 in-depth interviews of women who suffered from moderately severe and severe depression:

**a) Medical Comorbidities:** Pre-existing medical conditions contribute to an elevated risk of depression in women, as they impede productivity and give rise to chronic physical and financial stress. Common conditions such as diabetes, hypertension, and osteoarthritis are closely linked to mental health issues. As one participant stated,

*"Since I developed diabetes*, *I have experienced symptoms of sadness that were not present before*. *These symptoms now affect my family life*.*"*[40 years old, Matric, 4 children]

**b) Social Influences:** The influence of the mother-in-law, strongly impacts women’s decision-making authority within the household. Factors such as living conditions, societal perceptions, and women’s decision-making abilities have a definite influence on their mental well-being. One participant shared,

*"Despite being married for over 10 years and having six children*, *the presence of my mother-in-law and my husband’s harsh behavior often leaves me feeling helpless and disappointed*. *Thoughts of self-harm frequently cross my mind*, *but I refrain from acting on them for the sake of my children*.*"* [43 years old, Primary education, 6 children]

**c) Financial Situation:** Women from lower socioeconomic backgrounds exhibit higher rates of depression. Financial limitations restrict a family’s ability to function optimally and consequently strain relationships. A participant expressed,

*"I have spent my entire life hoping for an improvement in our financial conditions*, *but now I have lost all hope*. *There is nothing that can change my circumstances*. *People in poverty have nowhere to go*.*"* [46 years, uneducated, 4 children]

**d) Family Dynamics:** Crowded living conditions and excessive number of family members could contribute to mental health problems, while power dynamics within the household further complicate the situation. On the other hand, in certain circumstances, a larger family structure facilitates the sharing of responsibilities. One participant shared her perspective, stating,

*“Living in a large extended family has its challenges*, *with so many voices and opinions*. *It can be overwhelming at times*, *causing tensions and disagreements*. *But on the other hand*, *it also means that there are more people to help and support each other when needed*, *which provides some relief and a sense of belonging*.*"* [46 years, uneducated, 4 children]

**e) Number of Children:** Women with more than five children consistently experience high levels of stress, leading to not only depression but also anxiety. Financial constraints exacerbate these challenges, impairing their functioning and ability to positively influence their children’s lives. One woman expressed,

*"It is impossible for me to cope with stress all the time*. *These children are driving me crazy*. *Can you believe this interview is actually a break for me*? *I cannot think clearly when I am surrounded by seven screaming children*.*"* [28 years, Intermediate, 3 children]

**f) Recent Family Deaths:** Deaths within the immediate family, including parents, siblings, spouses, and children, had a profound impact on mental well-being. One participant shared,

*"I was managing my situation fairly well until my baby passed away last year*. *Since then*, *I have felt an overwhelming sadness that has paralyzed my life*. *Although I have three other children*, *the one I lost occupies most of my thoughts*. *Sometimes*, *I wish it had been me who died*.*"* [31 years, Primary education, 3 children]

**g) Marital Dissatisfaction:** Disturbed marital relationship emerged as a prominent theme influencing depression among married women. Conflict, lack of emotional support, and unfulfilled expectations within the marriage contributed to feelings of sadness and despair. As one participant explained,

*"My husband and I have grown distant over the years*. *We hardly communicate*, *and there is no emotional connection*. *It feels like I am living with a stranger*, *which often leaves me feeling depressed and isolated*.*"* [46 years, Bachelors, 4 children]

**h) Gender Roles and Societal Expectations:** The deeply ingrained norms and expectations placed on women to fulfill traditional roles as wives, mothers, and caregivers had a profound impact on their mental well-being. The pressure to conform to societal standards while simultaneously facing limited opportunities for personal growth and self-expression created a sense of dissatisfaction and frustration. One participant shared her experience, saying,

*"As a woman*, *I am expected to be the perfect wife*, *mother*, *and homemaker*, *always putting others’ needs before my own*. *It feels suffocating at times*, *like I’ve lost my own identity in the process*. *This constant pressure to meet society’s expectations has taken a toll on my mental health*, *leaving me feeling trapped and unfulfilled*.*"* [23 years, Intermediate, 1 child]

## Discussion

The findings of this research study indicated that factors such as age, financial situation, years of schooling, and recent deaths in the family were significantly associated with depression in this particular population. Our results are consistent with an older study that also found a correlation between increasing age of women and depression [19]. Perceived level of stress has been found higher among women as compared to men, that too compounded by low education, poverty and unemployment [20]. In accordance with other studies showed that there is a correlation between the increasing age of women and the occurrence of depression [21]. It is well-established that women belonging to low socioeconomic groups are more prone to suffer from depression [22]. This fact is also supported by our study findings. Major life circumstances, such as the death of a family member, can be significant stressors that contribute to poor mental health. Our study revealed a notable increase in depression among women who had undergone the death of a family member, providing additional support for the connection between bereavement and depression. Earlier research has similarly demonstrated a positive correlation between depression and the recent death of a family member [23]. Women often experience emotional exhaustion, whereas men may lean towards feelings of depersonalization. In fact, women appear to face a higher risk of psychological issues due to a blend of biological and social factors, including gender stereotypes, inequality, social isolation, and lack of autonomy [24].

Maternal depression has shown serious implications on children’s development and hence should be targeted in future programme planning [25]. This calls for an appropriate support and intervention for individuals coping with such experiences. The more social support the pregnant woman receives, the lower is her risk of depression [26]. Teaching coping strategies to the suffering women has shown positive effects [27]. Integration of health counseling into postnatal care and a non-judgmental service provision can help women improve their health in the postpartum period [28]. In our study, participants who were identified to be more depressed were referred for counseling by the principal investigator. This highlights the significance of mental health support and intervention for individuals experiencing depression. Referral to counseling services can provide individuals with the necessary tools and resources to manage their symptoms and improve their overall well-being [29]. Identifying mental health challenges in women, along with providing education, training, and interventions at different levels, would enhance the overall well-being of women’s mental health [30].

### Limitations of this study

While this study on women of reproductive age in post-COVID-19 times in Pakistan is the first of its kind, it is important to note that other significant determinants, such as family history of depression, current illness, recent death of parents, coping styles, and religious practices, could have provided a more comprehensive understanding of the issue. Representativeness of the sample of women in this study could be one of the limitation because these women represent only slums of Islamabad, and not the larger general population. Future research must take into account these variables and confounders for even better understanding of this public health problem.

## Conclusion

Depression is a commonly overlooked issue that can have both mental and physical symptoms. However, it disproportionately affects women, highlighting the need for increased monitoring in this population through regular screening programs aimed at early identification of depression. The approach to depression should shift from being solely focused on psychiatric treatment to a more preventive approach, involving family physicians. By integrating mental health screening and support into routine healthcare practices, we can work towards reducing the burden of depression and improving overall well-being for individuals, particularly women, affected by this condition.

## Supporting information

S1 Checklist (DOCX)

S1 Text Patient Health Questionnaire (PHQ9) used for collecting quantitative data. (DOCX)
