# Supplementary material for: Are social determinants associated with depression among married women of reproductive age? A mixed methods study from urban slums of Islamabad, Pakistan
Source: PLOS Glob Public Health. 2024 Jul 23;4(7):e0003463. doi: 10.1371/journal.pgph.0003463 (PMC11265670; doi:10.1371/journal.pgph.0003463)
Supplement: S1 Text — (DOCX) [file pgph.0003463.s002.docx]

**In-depth Interview Guide**

**Social determinants associated with depression among married women of reproductive age**

Q1. Are you suffering from any illness for which you are taking medications these days? (Probe any medical conditions for which she is under treatment, or any recent pat medical treatment, etc.)

Q2. How do you explain your decision making abilities when it is about your ‘health’? (Probe about her autonomy about decision making for treatment seeking, any person in family having the most influence, etc.)

Q3. Tell us something about your sources of income, and how do you cope with your household expenditures? (Probe: Financial autonomy, Household budget, Sources of income etc.)

Q4. What is your family structure, joint or nuclear? How many people live in this house? (Probe about family dynamics, relations, privacy, etc.)

Q5. How many children do you have? Are you satisfied with their upbringing? (Probe on coping with this number of children with financial constraints, quality of life etc.)

Q6. Was there any recent family tragedy/accident/demise? How did it affect your routine life? (Probe about affects specifically on mental health, she had faced because of this tragedy)

Q7. How do you describe your marital life, and how it has affected your overall well-being? (Probe about any issues with husband, in-laws, etc.)

Q8. How do you explain gender roles in your family? (Probe on responsibilities, division of labor, pressures, expectations, etc.)
